# Inter- and intra-tumoral heterogeneity on [^68^Ga]Ga-DOTA-TATE/[^68^Ga]Ga-DOTA-TOC PET/CT predicts response to [^177^Lu]Lu-DOTA-TATE PRRT in neuroendocrine tumor patients

**DOI:** 10.1186/s41824-024-00227-3

**Published:** 2024-11-30

**Authors:** Camila Gadens Zamboni, Ayca Dundar, Sanchay Jain, Marc Kruzer, Bradley T. Loeffler, Stephen A. Graves, Janet H. Pollard, Sarah L. Mott, Joseph S. Dillon, Michael M. Graham, Yusuf Menda, Ahmad Shariftabrizi

**Affiliations:** 1 Division of Nuclear Medicine, Department of Radiology-Room 3820-AJPP, University of Iowa Carver College of Medicine, 200 Hawkins Drive, Iowa City, IA 52242 USA; 2 Biostatistics Core, Holden Comprehensive Cancer Center, Iowa City, IA USA; 3 MIM Software Inc, Beachwood, OH USA; 4 Department of Medicine-Hematology/Oncology, University of Iowa Carver College of Medicine, Iowa City, IA USA

## Abstract

**Background:**

Indices of tumor heterogeneity on somatostatin receptor PET/CT scans may potentially serve as predictive biomarkers of treatment efficacy in neuroendocrine tumor (NET) patients undergoing [^177^Lu]Lu-DOTA-TATE PRRT.

**Methods:**

NET patients who underwent [^177^Lu]Lu-DOTA-TATE therapy at the University of Iowa from August 2018 to February 2021 were retrospectively evaluated. Radiomic features on the pre-PRRT somatostatin receptor PET/CT were evaluated using a custom MIM Software® LesionID workflow. Conventional PET/CT metrics of tumor burden, such as somatostatin receptor expression and tumor volume, were calculated in addition to the indices of tumor heterogeneity for each lesion (intra-lesional) and then summarized across all lesions throughout the body (inter-lesional). Endpoints included post-PRRT 24-month time to progression (TTP) and overall survival (OS). Cox regression models were used to assess the predictive ability of the imaging factors on post-PRRT 24-month TTP and OS. LASSO-penalized Cox regression was used to build a multivariable model for each outcome.

**Results:**

Eighty patients with a mean age of 65.1 years were included, with most (71.3%) completing 4 cycles of PRRT. Median TTP was 19.1 months, and OS at 60 months was 50%. A large degree of variability between patients was evidenced for imaging features related to somatostatin receptor expression. On multivariable analysis, total receptor expression and mean liver-corrected SUVmean were selected for 24-month TTP. The model was not able to significantly predict progression (C-statistic = 0.58, 95% CI 0.50–0.62). Total receptor expression and mean skewness were selected for OS. The resulting model was able to significantly predict death (C-statistic = 0.62, 95% CI 0.53–0.67), but the predictive ability was limited, as evidenced by the low C-statistic.

**Conclusions:**

Our exploratory analysis provides preliminary results showing that imaging indices of inter- and intra-tumor heterogeneity from pretreatment PET/CT images may potentially predict treatment efficacy in NET patients undergoing [^177^Lu]Lu-DOTA-TATE therapy. However, prospective evaluation in a larger cohort is needed to further assess whether a comprehensive characterization of tumor heterogeneity within a patient can help guide treatment decisions.

**Supplementary Information:**

The online version contains supplementary material available at 10.1186/s41824-024-00227-3.

## Introduction

Peptide Receptor Radionuclide Therapy (PRRT) consists of the systemic administration of radiolabeled somatostatin peptide analogs that specifically bind to overexpressed receptors in neuroendocrine tumor cells to promote highly targeted radiotherapy (Essen et al. 2009). PRRT with [^177^Lu]Lu-DOTA-TATE has been used in Europe in the past two decades. It was initially approved by the FDA in 2018 for the treatment of somatostatin receptor (SSTR) positive gastroenteropancreatic neuroendocrine tumors (NETs) (https://www.accessdata.fda.gov/drugsatfda_docs/label/2018/208700s000lbl.pdf). PRRT offers a new therapeutic option for patients with locally advanced or metastatic disease who progressed on first-line therapy with somatostatin analogs (SSAs) (Strosberg et al. 2017; Kendi et al. 2019; Howe 2022).

Although PRRT is a major step forward in the management of patients with advanced neuroendocrine tumors, its therapeutic efficacy is variable and difficult to predict (Laudicella et al. 2022). Extensive efforts have been made to determine the reasons for PRRT failure or suboptimal response in patients who have somatostatin avid lesions on [^68^Ga]Ga-DOTA-TATE scans (Laudicella et al. 2022; Gabriel et al. 2019; Bezzi et al. 2021). Several factors, such as elevated inflammatory markers, the presence of ascites, extensive liver metastases burden, multiple osseous metastases, and the presence of FDG avid disease, are known to be associated with poor response to PRRT (McClellan et al. 2022). Conversely, higher uptake on [^68^Ga]Ga-DOTA-TATE PET scans is associated with better response (McClellan et al. 2022; Kulkarni and Baum 2014). However, the available information in this regard does not yet allow the precise selection of patients for PRRT and accurate prognostication.

Tumor heterogeneity is a multifaceted and complex phenomenon increasingly recognized as a cause of cancer progression and treatment failure. It can be grossly categorized into inter-lesional tumor heterogeneity (variability between lesions in the body) or intra-lesional tumor heterogeneity (variability within a lesion). Inter-tumor heterogeneity refers to the presence of diverse subgroups in different metastatic lesions caused by multilayered factors, including genetic, cellular/molecular, functional, and histopathologic alterations, as well as anatomic location in a single patient (Pedraza-Arévalo et al. 2018; Lou et al. 2022; Yancovitz et al. 2012). Multilayer tumor heterogeneity, extending from the location of the tumor to the clinical and functional features, cellular signaling, genetic components, and molecular signature of the tumor, is a known phenomenon in NETs (Pedraza-Arévalo et al. 2018; Varghese et al. 2023). Intra- and inter-tumor heterogeneity are also clear causes of resistance to therapy and progression in NETs (Varghese et al. 2023; Reccia et al. 2023), such as in the case of spatiotemporal tumor heterogeneity in pancreatic cancer (Lou et al. 2022).

Imaging biomarkers have been applied with promising results in various diseases to predict response to treatment (Henry et al. 2022). Radiomics is an emergent data-driven field that enables the extraction of useful biomarkers from radiological images (Tomaszewski and Gillies 2021 May). It has been investigated as a surrogate for tumor heterogeneity in prior studies, as it is practically impossible to sample all metastatic tumor lesions in a patient (Reginelli et al. 2021; Bailly et al. 2019).

In the current study, inter- and intra-lesional tumor radiomic heterogeneity indices from pre-therapy [^68^Ga]Ga-DOTA-TATE/ [^68^Ga]Ga-DOTA-TOC scans were evaluated as potential biomarkers of treatment efficacy in patients with NETs undergoing PRRT.

## Materials and methods

### Patient selection

The University of Iowa Institutional Review Board approved this study (IRB# 200903778). All NET patients that received [^177^Lu]Lu-DOTA-TATE PRRT at our institution between 5/31/2018 and 2/24/2021 were retrospectively evaluated. A total of 99 patients were initially retrieved from the picture archiving and communication system (PACS) database. Nineteen patients were excluded, yielding 80 subjects for analysis. The reasons for exclusion were prior Y-90 PRRT (n = 8), unavailable/non-evaluable pre-therapy [^68^Ga]Ga-DOTA-TATE/[^68^Ga]Ga-DOTA-TOC imaging or taken ≥ 1 year before the beginning of [^177^Lu]Lu-DOTA-TATE PRRT (n = 10), and presence of only one lesion (n = 1).

### Imaging acquisition and radiopharmaceutical dose

Among the 80 pretreatment PET/CT scans, 59 were performed in-house. All in-house images used 3-D and time of flight modes. CT was performed for anatomic correlation and attenuation correction. PET, CT, and fused PET-CT images were reconstructed in axial, coronal, and sagittal planes. All in-house scanners met the minimum EARL requirements for recovery coefficients; however, this information was not available for outside studies. Mean injected radiopharmaceutical dose was 185 Megabecquerels (± 0.59 (SD) Megabecquerels). The mean uptake time was 63 min (± 13.1 min (SD)). Supplementary File-1 provides the detailed PET acquisition parameters and reconstruction methods, radiopharmaceutical dosing parameters, and CT acquisition data for all patients.

### Imaging processing and segmentation

Lesion segmentation and histogram-based features were calculated using a specifically designed semi-automatic workflow, “Ga-68 Dotatate Lesion ID” (MIM Software Inc., Cleveland, OH). The workflow automatically detects the lesions using the gradient-based methodology, and the investigator subsequently verifies lesions to eliminate physiologic sites of radiotracer uptake or to check the accuracy of contours. In this project, the investigator segmented any lesions that were missing from the automatic segmentation using PETEdge and PETEdge+ tools based on SUV gradients rather than rigid numerical cut-off values. This gradient-based methodology (Geets et al. 2007) is a well-established method (Werner-Wasik et al. 2012; Takeda et al. 2017; Liberini et al. 2021; Ha et al. 2019) of segmentation, and it has been shown to be a better alternative compared to manual segmentation (Ha et al. 2019). Gradient-based tumor segmentation has been shown to be more precise compared to thresholding methods, which are known to underestimate the tumor volume and are confounded by variations in contrast and noise (Wanet et al. 2011). A nuclear medicine physician (A.S.) independently re-evaluated all PET/CTs. Two investigators (A.S. & J.P., both with 5+ years of experience in the field) reviewed and agreed on questionable lesions regardless of the original clinical interpretation of the images.

The MIM workflow generated the following imaging parameters: volume (ml), skewness, kurtosis, SUVmax, SUVmean, standard deviation of SUV (SDSUV), liver SUVmean, and liver SUVmax. For each patient, all lesions were included when calculating the total volume and number of lesions. For the remaining imaging indices, lesions with a volume of less than 0.524 cc (metabolic volume) were excluded to avoid a partial volume effect (Soret et al. 2007; Wallstén et al. 2013).

### Imaging predictors

Table 1 outlines the intra-lesional and inter-lesional metrics, specifies the source (software generated vs. calculated), lists the formula for calculated variables, and references the literature that has utilized such indices. Intra-lesional metrics included SUVmean, SUVmax, SDSUV, skewness, kurtosis, volume, coefficient of variation of SUV (CVSUV), SUVmax/SUVmean, (SUVmax–SUVmean)/SUVmean, receptor expression, liver-corrected receptor expression, liver-corrected SUVmean, and two versions of liver-corrected SUVmax. Given that the outcomes of interest were captured at the patient level, the intra-lesional indices of tumor heterogeneity had to be reduced from the lesion level to the patient level. Each intra-lesional index was summarized across all lesions within a patient using the mean, maximum (max), and standard deviation (SD) functions. For example, the max SUVmean is the largest SUVmean across all lesions within the patient. To quantify tumor extent within a patient’s body, the following indices were summarized using the total function to obtain inter-lesional metrics: tumor volume, number of lesions, receptor expression, and liver-corrected receptor expression. Inter-lesional metrics also included max SUVmax/min SUVmax, max SUVmax–min SUVmean, max (SUVmax–SUVmean), average tumoral heterogeneity, and maximal tumor divergence. The presence of liver-dominant disease was determined by comparing the total tumor volume in the liver to the total tumor volume in the body.

**Table 1:** Indices of intra and inter-tumor heterogeneity, the source, the formula for calculated indices and references for historical literature utilizing the indices

Indices	Source	Calculation	References
*Intra-Lesional*^a^			
SUVmean	Software		Ortega et al. (2021)
SUVmax	Software		Ortega et al. (2021)
Standard Deviation of SUV (SDSUV)	Software		
Skewness	Software		Ortega et al. (2021), Önner et al. (2020), Laudicella et al. (2022), Atkinson et al. (2021)
Kurtosis	Software		Ortega et al. (2021), Önner et al. (2020), Laudicella et al. (2022), Atkinson et al. (2021)
Volume	Software		Tirosh et al. (2018)
Coefficient of Variation SUV (CVSUV)	Calculated	SDSUV/SUVmean	Bundschuh et al. (2014)
SUVmax/SUVmean	Calculated	SUVmax/SUVmean	Gong et al. (2022)
(SUVmax–SUVmean)/SUVmean	Calculated	(SUVmax–SUVmean)/SUVmean	Xie et al. (2019)
Receptor Expression	Calculated	Volume x SUVmean	Kim et al. (2020), Toriihara et al. (2019)
Liver-Corrected Receptor Expression	Calculated	Volume x (SUVmean/Liver SUVmean)	
Liver-Corrected SUVmean	Calculated	SUVmean/Liver SUVmean	
Liver-Corrected SUVmax	Calculated	SUVmax/Liver SUVmax	Kim et al. (2020)
Liver-Corrected SUVmax (lmean)	Calculated	SUVmax/Liver SUVmean	Toriihara et al. (2019)
*Inter-Lesional*			
Total Volume	Calculated	Sum of all lesions	Tirosh et al. (2018)
Number of Lesions	Calculated	Count of # of lesions	
Total Receptor Expression	Calculated	Sum of receptor expression of all lesions	
Total Liver-Corrected Expression	Calculated	Sum of liver-corrected expression of all lesions	
Max SUVmax/Min SUVmax	Calculated	Max SUVmax/Min SUVmax	Gong et al. (2018)
Max SUVmax–Min SUVmean	Calculated	Max SUVmax–Min SUVmean	Xie et al. (2022)
Max (SUVmax–SUVmean)	Calculated	Max (SUVmax–SUVmean)	Xie et al. (2022)
Average Tumoral Heterogeneity	Calculated	Average of pairwise cosine dissimilarities across all lesions	Sun et al. (2022)
Maximal Tumor Divergence	Calculated	Maximum of pairwise cosine dissimilarities across all lesions	Sun et al. (2022)
Liver Dominant Disease	Calculated	Yes, if total volume of liver lesions > total volume of all lesions within a patient	

^a^Each of the intra-lesional variables were summarized across all lesions within a patient using the mean, maximum, and standard deviation functions

### [^177^Lu]Lu-DOTA-TATE PRRT

[^177^Lu]Lu-DOTA-TATE PRRT was performed according to the manufacturer's guidelines. Two patients received part of their treatment at the local Veteran Affairs Hospital following the same guidelines. Dose adjustment was performed according to the package insert when clinically necessary. The number of PRRT cycles received was also included as one of the candidate predictors (1–2 cycles vs. 3–4 cycles).

### Adverse events

Kidney, liver, and hematological function tests were collected at baseline (within 3 months of the beginning of the therapy), in between each cycle, immediately after the last cycle (between 20 and 40 days after therapy), and between 3 and 9 months from the end of therapy. Laboratory results were graded according to the Common Terminology Criteria for Adverse Events (CTCAE, version 5.0). In addition to these biochemical markers, the presence or absence of the following symptoms was recorded to evaluate adverse events: nausea/vomiting, abdominal pain, diarrhea, fatigue/tiredness, weight loss, flushing, wheezing, musculoskeletal/joint pain, and hemodynamic status change. The hemodynamic status change was defined as hypotension/hypertension or tachycardia/bradycardia, as mentioned in the clinic note.

### Statistical analysis

Survival probabilities were estimated and plotted using the Kaplan–Meier method. Estimates, along with 95% pointwise confidence intervals, are reported. For 24-month time to progression (TTP), time was calculated from the last cycle of [^177^Lu]Lu-DOTA-TATE PRRT treatment to clinical or radiological progression within 24 months, whichever occurred first. Clinical (“real-world”) progression (Almeamar et al. 2022; Griffith et al. 2019; Torres et al. 2022; Morse et al. 2020) was defined as (1) therapy change (newly prescribed or increase in dose or frequency of a somatostatin analog long-acting release (SSA-LAR) or antineoplastic or chemotherapeutic agents), (2) worsening symptoms or laboratory markers, (3) clinician’s interpretation of imaging results, (4) new biopsy results, or (5) cancer-related death. Radiological progression was defined as evidence of an increase in disease burden, which was determined by systematic analysis of imaging reports or application of RECIST v1.1 criteria to lesions on cross-sectional images (contrasted MRI or CT of the abdomen, pelvis, or chest) (Eisenhauer et al. 2009). Images done 3 months prior to the last cycle were discarded due to the increased chance of pseudo-progression. Otherwise, patients were censored at the last disease evaluation or 24 months, whichever occurred first. Twenty-four-month TTP was chosen based on the clinically relevant time point of mid-term response evaluation per the NETTER-1 trial methodology (Strosberg et al. 2017). For OS, time was calculated from the last cycle of [^177^Lu]Lu-DOTA-TATE PRRT PRRT treatment to death due to any cause. Patients who were still alive were censored at the date last known to be alive. Cox regression models were used to assess the predictive ability of imaging variables for 24-month TTP and OS. Estimated effects of imaging predictors are reported as hazard ratios (HR) along with 95% confidence intervals (CI), and predictive performance was estimated using Uno’s C-statistic and compared to a null value of 0.50, which indicates the model discriminates patients with events from patients without events no better than chance.

Given the number of imaging predictors under consideration, a LASSO-penalized Cox regression was utilized for each outcome to build a multivariable model and identify important predictors. One thousand iterations of threefold cross-validation were employed to determine the value of LASSO’s penalty term, lambda, which provides the highest average C-statistic for the Cox regression models. Optimism-corrected C-statistics, along with 95% CIs, were constructed for each multivariable model using 1,000 bootstrap samples. Multivariable models in which the 95% confidence interval excluded 0.50 were able to predict the outcome better than chance. All tests were two-sided and assessed for significance at the 5% level using SAS v9.4 (SAS Institute, Cary, NC) and R (www.r-project.org).

## Results

### Patient characteristics

Eighty patients met the inclusion criteria and were included in the final analysis. The mean age was 65.1 years (Table 2). Most patients had a midgut NET primary (72.9%) in the ileum (61.8%). Grade 2 tumors (61.3%) with Ki-67 at 3–20% (69.0%) were the most prevalent.

**Table 2:** Baseline patient, disease, and treatment characteristics

Characteristics	N = 80 (%)
Mean age ± SD, years (range)	65.1 ± 11.3 (23–89)
Mean BMI ± SD (range)	28.4 ± 7.3 (16.1–52.0)
Sex	
Female	36 (45.0)
Male	44 (55.0)
Race	
Non-White	4 (5.0)
White	76 (95.0)
Primary NET Type	
Foregut	18 (25.7)
Midgut	51 (72.9)
Hindgut	1 (1.4)
Missing	10
Primary NET Location	
Lung	5 (7.4)
Pancreas	12 (17.6)
Ileum	42 (61.8)
Small Bowel	6 (8.8)
Large Bowel	2 (2.9)
Other Organ	1 (1.5)
Missing	12
Has Liver Lesions?	73 (91.3)
Has Bone Lesions?	36 (45.0)
Has Nodal Lesions?	55 (68.8)
Has Soft Tissue Lesions?	50 (62.5)
Ki-67 Index (Most Advanced)	
< 3%	16 (22.5)
3–20%	49 (69.0)
> 20%	6 (8.5)
Missing	9
Tumor Grade (Most Advanced)	
G1	15 (20.0)
G2	46 (61.3)
G3	5 (6.7)
Well-differentiated	9 (12.0)
Missing	5
Number of Cycles Completed	
1	5 (6.3)
2	5 (6.3)
3	13 (16.3)
4	57 (71.3)

### Prior therapeutic regimens

Prior to [^**177**^Lu]Lu-DOTA-TATE PRRT, 69 (86.3%) patients underwent surgery on a primary tumor or metastasis. Fifty (62.5%) patients received at least one type of therapeutic regimen, including systemic chemotherapy (n = 12), immunotherapy including interferon (n = 1), sunitinib (n = 2), everolimus (n = 21), chemoembolization (n = 3), bland embolization (n = 13), ablation including Y-90 microsphere (n = 6), or RFA/microwave (n = 18). Seventy-five (93.8%) patients had received somatostatin analogs, including octreotide or lanreotide.

### Imaging

Of the 80 patients included, 75 underwent [^68^Ga]Ga-DOTA-TATE PET/CT, and 5 underwent Ga-68 DOTATOC PET/CT scans prior to therapy. Fifty-nine pre-therapy scans were performed in-house, while 21 were external to our institution. The 5 [^68^Ga]Ga-DOTA-TOC PET/CT scans were performed at our institution. [^68^Ga]Ga-DOTA-TOC was produced locally and used for PET/CT before DOTA-TATE became commercially available.

### [^177^Lu]Lu-DOTA-TATE PRRT

Fifty-seven (71.3%) patients completed all 4 cycles of planned treatment (Table 2). Reasons for treatment discontinuation were hematologic laboratory abnormalities, dosimetry results, loss at follow-up, or any cause of worsening medical condition.

### Adverse events

The incidence of laboratory adverse events was limited. Grade 3 thrombocytopenia, anemia, and increased bilirubin, ALT, and Grade 4 creatinine were evidenced in one patient each (Table 4). The most common adverse symptoms were fatigue/tiredness (72.5%), diarrhea (47.5%), nausea/vomiting (43.8%), and abdominal pain (42.5%, Table 3).

**Table 3:** Summary of the adverse events observed in the study population

Adverse event	N = 80
Blood Disorders	
Grade 3 or 4 thrombocytopenia	1 (1.3)*
Grade 3 or 4 anemia	1 (1.3)*
Grade 3 or 4 leukopenia	0 (0)
Grade 3 or 4 neutropenia	0 (0)
Liver Function Tests	
Grade 3 or 4 hypoalbuminemia	0 (0)
Grade 3 or 4 bilirubin increase	1 (1.3)*
Grade 3 or 4 ALT increase	1 (1.3)*
Grade 3 or 4 AST increase	0 (0)
Urinary	
Grade 3 or 4 creatinine increase	1 (1.3)*
Gastrointestinal Disorders	
Nausea/vomiting	35 (43.8)
Abdominal pain	34 (42.5)
Diarrhea	38 (47.5)
General Disorders	
Fatigue/tiredness	58 (72.5)
Weight loss	10 (12.5)
Vascular Disorders	
Flushing	23 (28.8)
Respiratory	
Wheezing	2 (2.5)
Musculoskeletal Disorders	
Musculoskeletal/joint pain	22 (27.5)
Other	
Hemodynamic status change	5 (6.3)

Subjective treatment-related toxicities and grade 3 and 4 laboratorial (hematologic, liver, and renal markers) abnormalities are reported

*Instances of thrombocytopenia, anemia, bilirubin increase, and ALT increase were Grade 3. The instance of creatinine increase was Grade 4

### 24-Month TTP and OS

**Fig. 1:**
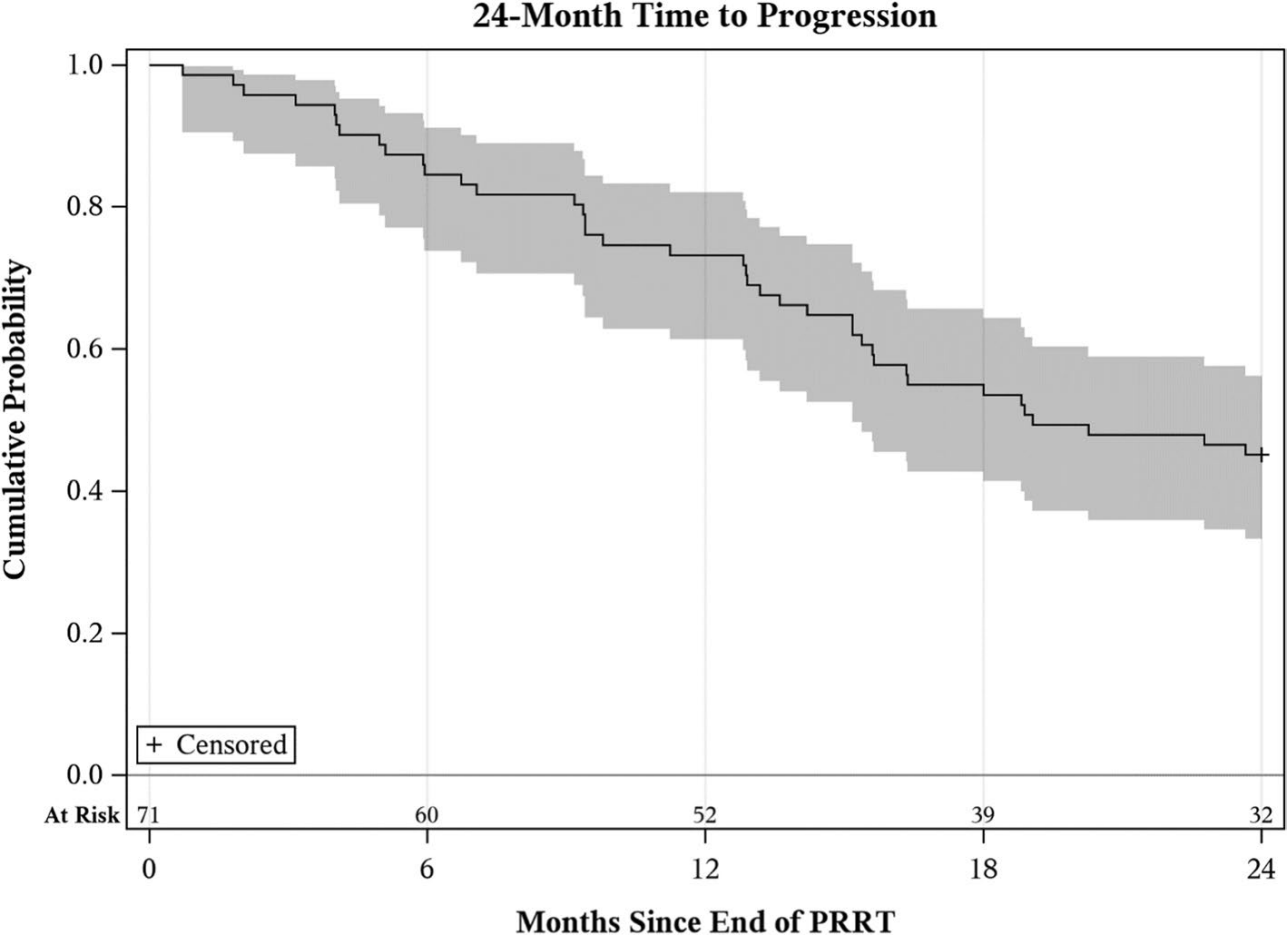
Kaplan–Meier curve for 24-Month time to progression (TTP) post-PRRT. TTP at 12 and 24 months was 73% (95% CI 61–82%) and 45% (95% CI 33–56%), respectively

**Fig. 2:**
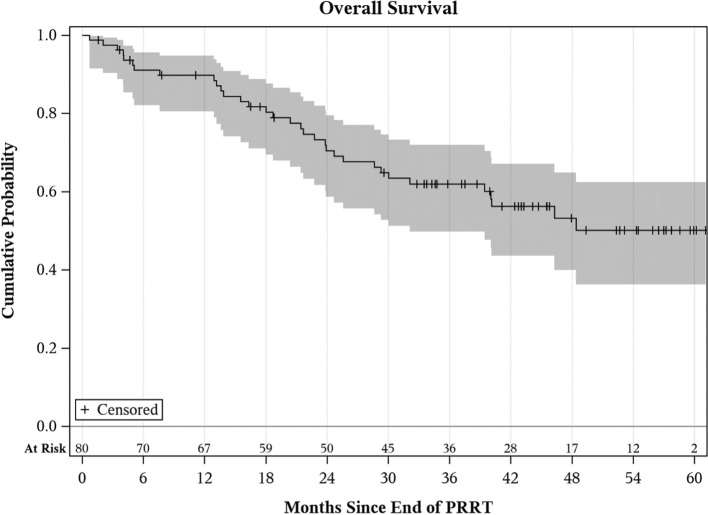
Kaplan–Meier curve for overall survival (OS) post-PRRT. OS at 12, 24, and 60 months was 90% (95% CI 81–95%), 70% (95% CI 59–80%), and 50% (95% CI 36–62%), respectively

Of the 80 patients included, 71 patients were included in the 24-month TTP analysis. The remaining 9 patients did not have data available for evaluation of progression after PRRT at this time point. Within the 24-month TTP period, 39 patients experienced a clinical or imaging progression, and 10 of these patients died. The median TTP was 19.1 months (Fig. 1). TTP rates were 73% (95% CI 61–82%) and 45% (95% CI 33–56%) at 12 and 24 months, respectively. At a median survival follow-up of 34 months (range 0.7–61.1 months), 33 patients had died, and median OS had not been reached (Fig. 2). OS rates were 90% (95% CI 81–95%), 70% (95% CI 59–80%), and 50% (95% CI 36–62%) at 12, 24, and 60 months, respectively.

### Lesions and imaging features

**Fig. 3:**
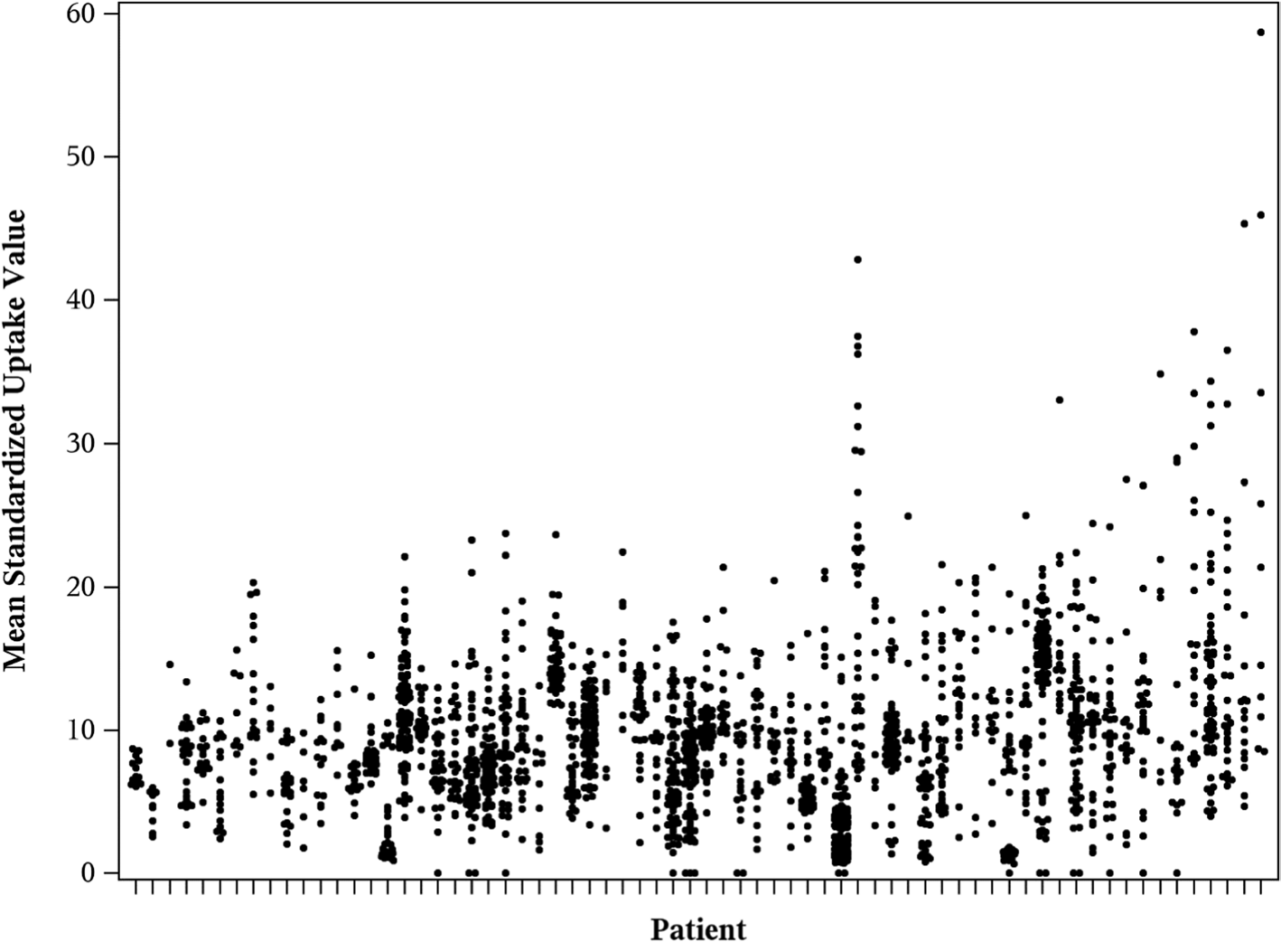
Inter-lesional variability of SUVmean for each patient

**Fig. 4:**
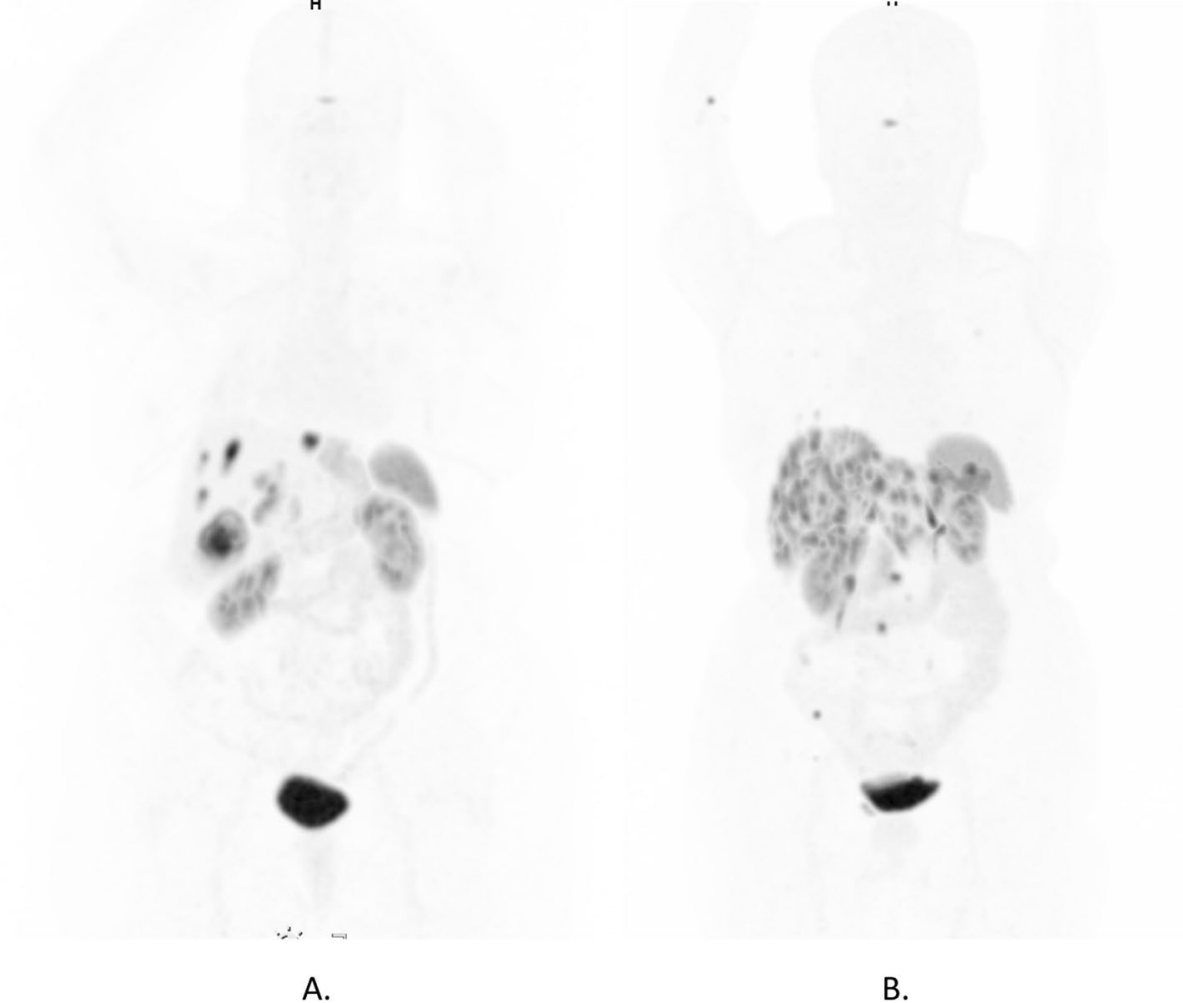
Representative PET/CTs of patients with high and low inter-tumor heterogeneity. **A** [^68^Ga]Ga-DOTA-TATE PET/CT of patient with calculated SD SUVmean of 11.4 (high inter-tumor heterogeneity). **B** Ga-68 DOTATOC PET/CT of patient with SD SUVmean of 0.94 (low inter-tumor heterogeneity)

A total of 3214 lesions (1241 liver, 1110 bone, 435 nodal, and 428 soft tissue) were initially identified, and after applying the volume criteria, 2122 lesions (1053 liver, 540 bone, 283 node, and 246 soft tissue) were included in the relevant imaging metrics. Imaging features are descriptively summarized in Supplemental Table 1. For some imaging features, more variability (CV > 1.0) between patients was evidenced, which was especially the case for imaging features related to somatostatin receptor expression. Figure 3 shows the inter-lesional variability in the mean uptake for each lesion within a patient. Figure 4 shows representative somatostatin receptor PET/CTs of two patients with high and low inter-tumor heterogeneity.

### 24-Month TTP

Among the 53 quantitative imaging features, along with liver dominant disease and number of PRRT cycles, the following indices were significantly predictive of 24-month TTP on univariable analysis (Table 4): mean receptor expression (C-statistic = 0.60, p = 0.04), total liver-corrected receptor expression (C-statistic = 0.61, p = 0.04), mean liver-corrected receptor expression (C-statistic = 0.62, p = 0.01), and SD liver-corrected receptor expression (C-statistic = 0.61, p = 0.05). On multivariable analysis, total receptor expression and mean liver-corrected SUVMean were included in the model (Table 5). The selected model had an optimism-corrected C-statistic of 0.58 (95% CI 0.50–0.62), which was not significantly better at predicting 24-month TTP than chance alone.

**Table 4:** Imaging features significantly predictive of 24-Month TTP and OS on univariable analysis

Outcome	Predictor	N	HR direction	HR	95% CI		C-Statistic	P-value
24-Month TTP	Mean Receptor Expression	71	+	1.00	1.00	1.00	0.60	**0.04**
	Total Liver-Corrected Receptor Expression	71	+	1.00	1.00	1.00	0.61	**0.04**
	Mean Liver-Corrected Receptor Expression	71	+	1.00	1.00	1.00	0.62	**0.01**
	SD Liver-Corrected Receptor Expression	71	+	1.00	1.00	1.00	0.61	**0.05**
OS	Mean Skewness	80	–	0.19	0.05	0.67	0.63	**0.02**

The direction of the hazard ratio is reported as follows: (+) indicates that increasing values are associated with decreased 24-Month TTP/OS and (−) indicates that increasing values are associated with increased 24-Month TTP/OS

**Table 5:** Multivariable models for predictors of 24-month TTP and OS

Variable	Outcome	
	24 Month TTP N = 71	OS N = 80
Total Receptor Expression	+	+
Mean Liver-Corrected SUVMean	+	
Mean Skewness		–
Concordance	0.61	0.66
Optimism Corrected Concordance (95% CI)	0.58 (0.50–0.62)	0.62 (0.53–0.67)

The direction of the hazard ratio is reported as follows: (+) indicates that increasing values are associated with decreased 24-Month TTP/OS and (−) indicates that increasing values are associated with increased 24-Month TTP/OS

### OS

On univariable analysis, only mean skewness (concordance = 0.63, p = 0.02) was significantly predictive of OS (Table 4). On multivariable analysis, total receptor expression and mean skewness were included in the model and had an optimism-corrected C-statistic of 0.62 (95% CI 0.53–0.67), which was significantly better at predicting overall survival than chance alone (Table 5).

## Discussion

Although lesion-based analysis of one or a few selected lesions is less time-consuming and simpler, it can potentially lead to misevaluation of inter-tumor heterogeneity (Henry et al. 2022; Sun et al. 2022), resulting in poor response assessment of metastatic disease due to the diverse number of lesions in various locations. Using a patient-level approach with segmentation that includes all lesions helps avoid this pitfall. In our study, we used previously published indices of inter- and intra-tumor heterogeneity that could be evaluated with PET imaging and introduced several new indices to summarize intra-tumor metrics across the body to better estimate inter-tumor heterogeneity of lesions in each patient. To the best of our knowledge, few reports have previously evaluated the role of intra- and inter-tumor heterogeneity in NET patients undergoing [^177^Lu]Lu-DOTA-TATE PRRT.

Intra-tumor heterogeneity refers to different subgroups of tumor cell types within a single lesion at genetic and histopathologic levels (Liu et al. 2022). Several studies have investigated the association between intra-tumor heterogeneity indices and treatment outcomes in different diseases. Radiomics is considered a robust approach for non-invasively evaluating intra-tumor heterogeneity, and several studies have shown an association between high tumor heterogeneity and worse outcomes in various cancer types. For example, higher heterogeneity of skewness in MRI-based radiomics was associated with greater benefit from neoadjuvant chemotherapy in locally advanced rectal cancer (Coppola et al. 2021). In another study, higher intra-tumor metabolic heterogeneity quantified by the AUC-CSH index on FDG PET/CTs in patients with neuroblastomas was associated with worse treatment outcomes (Liu et al. 2022). In the case of NETs, however, only a few indices of intra-tumoral heterogeneity have been previously applied to somatostatin receptor PETs in these patients, such as heterogeneity of SSTR expression assessed by the coefficient of variation (CV) (Fonti et al. 2022). Other studies have used a qualitative visual index for this purpose (Kratochwil et al. 2015).

In contrast, many studies have investigated (mostly intra-tumor) heterogeneity on FDG PET/CT using calculated indices that we also included in this study. Overall, the general assumption is that NETs with more heterogenous somatostatin receptor expression should have a worse response to PRRT. Consistent with the above hypothesis, Graf et al. found that heterogenous SSTR expression on target lesions, based on visual inspection, was related to poorer TTP and OS (Graf et al. 2020 Apr). On the other hand, other studies using image-based parameters, such as skewness, kurtosis, and entropy, have reported inconsistent findings (Önner et al. 2020; Atkinson et al. 2021; Werner et al. 2019, 2017). A lesion-based radiomics analysis of 65 PET features for each lesion revealed that HISTO_Skewness and HISTO_Kurtosis were related to therapy response (Laudicella et al. 2022). Werner et al. demonstrated positive associations between entropy and OS and PFS and between skewness and OS in NET patients undergoing PRRT (Werner et al. 2019, 2017). In contrast, another study demonstrated that measures of heterogeneity, including higher kurtosis, higher entropy, and lower skewness, were related to worse PFS and OS in NET patients undergoing [^177^Lu]Lu-DOTA-TATE therapy (Gong et al. 2022). Finally, another study using a lesion-based analysis showed an association between lower skewness and kurtosis with better PRRT response (Atkinson et al. 2021).

On multivariable analysis, total receptor expression was included in both models for 24-month TTP and OS. In addition to total receptor expression, mean liver-corrected SUVmean was included in the 24-month TTP model and mean skewness in the OS model, respectively. Toriihara et al. found that increasing somatostatin receptor-expressing tumor volume but not total receptor expression was associated with poorer PFS in well-differentiated NET patients (Toriihara et al. 2019). Ortega et al. also reported an association between liver-corrected SUVmean and skewness with outcomes (Ortega et al. 2021). While the multivariable models in this study provided limited predictive performance, as evidenced by the C-statistics, the consistency of these results with the prior literature suggests that whole-body radiomic evaluation could be used to predict patient outcomes. The existing studies have used different analytical approaches, making the comparisons between the multivariable models difficult. In the current study, the multivariable models were built to maximize predictive performance (e.g., C-statistic), whereas existing literature has focused more on estimating an association (e.g., testing hazard ratios). As a post-hoc analysis, we conducted univariable analyses to evaluate the association between the selected features and each outcome. Supplemental Fig. 1 includes volcano plots, which plot log-transformed p-values against the estimated hazard ratio for each variable separately for TTP and OS. For 24-month TTP, features that showed a significant association included total receptor expression, max receptor expression, total liver-corrected receptor expression, mean liver-corrected receptor expression, and mean liver-corrected SUVmean all with positive hazard ratio direction; for OS, only total receptor expression and mean skewness demonstrated a significant association, with positive and negative hazard ratio directions, respectively.

Our study had several limitations that should be noted. Like in prior literature, the modest sample size and limited number of events may not have been sufficient to characterize the predictive performance of imaging features. In addition, this study was conducted at a single institution; therefore, our results may not be generalizable to other patient populations. Lastly, SUV harmonization was not performed across the images from different scanners and only SUV normalization was done for some parameters. Regarding this study’s strengths, all tumor lesions in each patient were segmented and included in the analysis, as opposed to selecting only a few lesions in each organ. We also focused on first-order radiomics that are less prone to variation amongst different scanners.

## Conclusion

Our exploratory analysis provides preliminary evidence showing that imaging indices of inter- and intra-tumor heterogeneity taken from pretreatment PET/CT images may predict treatment efficacy in NET patients undergoing [^177^Lu]Lu-DOTA-TATE therapy. However, prospective evaluation in a larger cohort is needed to further assess whether a comprehensive characterization of tumor heterogeneity within a patient can help guide treatment decisions.

## Supplementary Information

Supplementary Material 1.

## Data Availability

The data for this study can be shared upon reasonable request/proposal pending the approval of the University of Iowa’s Department of Sponsored Programs and Data Governance Committee.
