# Supplementary material for: Inter- and intra-tumoral heterogeneity on [68Ga]Ga-DOTA-TATE/[68Ga]Ga-DOTA-TOC PET/CT predicts response to [177Lu]Lu-DOTA-TATE PRRT in neuroendocrine tumor patients
Source: EJNMMI Rep. 2024 Nov 30;8(1):39. doi: 10.1186/s41824-024-00227-3 (PMC11607192; doi:10.1186/s41824-024-00227-3)
Supplement: Supplementary file 1 — Supplementary Material 1. [file 41824_2024_227_MOESM1_ESM.docx]

| **Variable** | **N** | **Min** | **Max** | **Median** | **Mean** | **SD** | **CV** |
| --- | --- | --- | --- | --- | --- | --- | --- |
| Number of Lesions | 80 | 2.0 | 250.0 | 25.5 | 40.2 | 44.2 | 1.1 |
| Average Tumoral Heterogeneity | 80 | 0.2 | 1.8 | 0.4 | 0.5 | 0.3 | 0.5 |
| Maximal Tumor Divergence | 80 | 0.4 | 2.0 | 1.5 | 1.5 | 0.5 | 0.3 |
| MaxSUVmax / MinSUVmax | 80 | 2.0 | 52.1 | 8.5 | 11.1 | 8.6 | 0.8 |
| MaxSUVmax - MinSUVmean | 80 | 8.4 | 256.1 | 42.6 | 50.1 | 34.8 | 0.7 |
| Max (SUVmax - SUVmean) | 80 | 5.6 | 216.9 | 28.9 | 37.2 | 30.1 | 0.8 |
| Total Receptor Expression | 80 | 184.6 | 29233.7 | 3521.9 | 5649.8 | 5855.2 | 1.0 |
| Mean Receptor Expression | 80 | 13.9 | 2434.1 | 167.6 | 342.4 | 479.1 | 1.4 |
| Max Receptor Expression | 80 | 40.3 | 26887.0 | 1979.7 | 4019.5 | 5167.5 | 1.3 |
| SD Receptor Expression | 80 | 12.0 | 7718.7 | 480.9 | 968.8 | 1348.9 | 1.4 |
| Total Liver-Corrected Receptor Expression | 80 | 40.5 | 6315.4 | 807.3 | 1385.9 | 1480.9 | 1.1 |
| Mean Liver-Corrected Receptor Expression | 80 | 2.8 | 756.0 | 34.5 | 85.6 | 127.9 | 1.5 |
| Max Liver-Corrected Receptor Expression | 80 | 11.4 | 5953.6 | 471.3 | 974.9 | 1260.9 | 1.3 |
| SD Liver-Corrected Receptor Expression | 80 | 3.2 | 1486.0 | 117.2 | 229.8 | 305.0 | 1.3 |
| Total Tumor Volume (ml) | 80 | 24.3 | 1932.5 | 269.0 | 380.6 | 375.6 | 1.0 |
| Mean Volume | 80 | 1.1 | 125.9 | 8.8 | 17.4 | 25.4 | 1.5 |
| Max Volume | 80 | 4.2 | 1728.4 | 132.5 | 251.9 | 333.1 | 1.3 |
| SD Volume | 80 | 1.2 | 375.2 | 26.0 | 52.2 | 71.8 | 1.4 |
| Mean Kurtosis | 80 | -0.5 | 2.2 | 0.2 | 0.2 | 0.5 | 2.1 |
| Max Kurtosis | 80 | -0.0 | 36.7 | 2.8 | 4.3 | 6.0 | 1.4 |
| SD Kurtosis | 80 | 0.3 | 5.4 | 1.0 | 1.2 | 0.9 | 0.8 |
| Mean SUVmax | 80 | 4.7 | 103.1 | 17.7 | 21.3 | 13.7 | 0.6 |
| Max SUVmax | 80 | 11.0 | 264.7 | 48.4 | 54.5 | 35.7 | 0.7 |
| Min SUVmax | 80 | 0.9 | 29.9 | 5.5 | 6.7 | 4.6 | 0.7 |
| SD SUVmax | 80 | 2.9 | 98.7 | 11.2 | 13.8 | 12.6 | 0.9 |
| Mean (SUVmax / SUVmean) | 80 | 1.3 | 3.5 | 1.8 | 1.8 | 0.4 | 0.2 |
| Max (SUVmax / SUVmean) | 80 | 1.7 | 5.9 | 3.2 | 3.3 | 0.9 | 0.3 |
| SD (SUVmax / SUVmean) | 80 | 0.1 | 1.6 | 0.5 | 0.6 | 0.3 | 0.5 |
| Mean SUVmean | 80 | 2.8 | 24.0 | 9.6 | 10.3 | 3.8 | 0.4 |
| Max SUVmean | 80 | 6.0 | 58.7 | 17.6 | 19.7 | 9.1 | 0.5 |
| Min SUVmean | 80 | 0.6 | 11.8 | 3.9 | 4.4 | 2.7 | 0.6 |
| SD SUVmean | 80 | 0.9 | 17.2 | 3.5 | 4.2 | 2.6 | 0.6 |
| Mean SUVSD | 80 | 0.5 | 21.7 | 2.6 | 3.5 | 2.9 | 0.8 |
| Max SUVSD | 80 | 1.5 | 63.7 | 8.3 | 10.0 | 8.2 | 0.8 |
| SD SUVSD | 80 | 0.4 | 23.0 | 2.1 | 2.8 | 2.8 | 1.0 |
| Mean Skewness | 80 | 0.0 | 1.6 | 0.7 | 0.7 | 0.3 | 0.4 |
| Max Skewness | 80 | 0.7 | 5.5 | 1.6 | 1.8 | 0.8 | 0.4 |
| SD Skewness | 80 | 0.2 | 1.0 | 0.5 | 0.5 | 0.2 | 0.3 |
| Mean SUVCV | 80 | 0.2 | 0.7 | 0.3 | 0.3 | 0.1 | 0.3 |
| Max SUVCV | 80 | 0.3 | 1.2 | 0.5 | 0.6 | 0.2 | 0.3 |
| SD SUVCV | 80 | 0.0 | 0.4 | 0.1 | 0.1 | 0.1 | 0.4 |
| Mean Liver-Corrected SUVmean | 80 | 0.6 | 7.4 | 2.3 | 2.6 | 1.2 | 0.5 |
| Max Liver-Corrected SUVmean | 80 | 1.5 | 14.6 | 4.1 | 4.9 | 2.6 | 0.5 |
| SD Liver-Corrected SUVmean | 80 | 0.3 | 3.6 | 0.9 | 1.0 | 0.7 | 0.7 |
| Mean Liver-Corrected SUVmax | 80 | 0.7 | 15.2 | 3.2 | 3.9 | 2.6 | 0.7 |
| Max Liver-Corrected SUVmax | 80 | 2.5 | 38.9 | 8.4 | 10.0 | 6.6 | 0.7 |
| SD Liver-Corrected SUVmax | 80 | 0.4 | 14.5 | 2.0 | 2.5 | 2.3 | 0.9 |
| Mean Liver-Corrected SUVmax (lmean) | 80 | 0.8 | 23.1 | 4.4 | 5.4 | 4.1 | 0.7 |
| Max Liver-Corrected SUVmax (lmean) | 80 | 2.8 | 56.5 | 12.2 | 13.7 | 9.7 | 0.7 |
| SD Liver-Corrected SUVmax (lmean) | 80 | 0.4 | 20.6 | 2.8 | 3.5 | 3.5 | 1.0 |
| Mean (SUVmax - SUVmean) / SUVmean | 80 | 0.3 | 2.5 | 0.8 | 0.8 | 0.4 | 0.4 |
| Max (SUVmax - SUVmean) / SUVmean | 80 | 0.7 | 4.9 | 2.2 | 2.3 | 0.9 | 0.4 |
| SD (SUVmax - SUVmean) / SUVmean | 80 | 0.1 | 1.6 | 0.5 | 0.6 | 0.3 | 0.5 |

**Supplemental Table 1.** Descriptive statistics of all 53 imaging predictors amongst 80 patients. SD=Standard Deviation; CV=Coefficient of Variation.


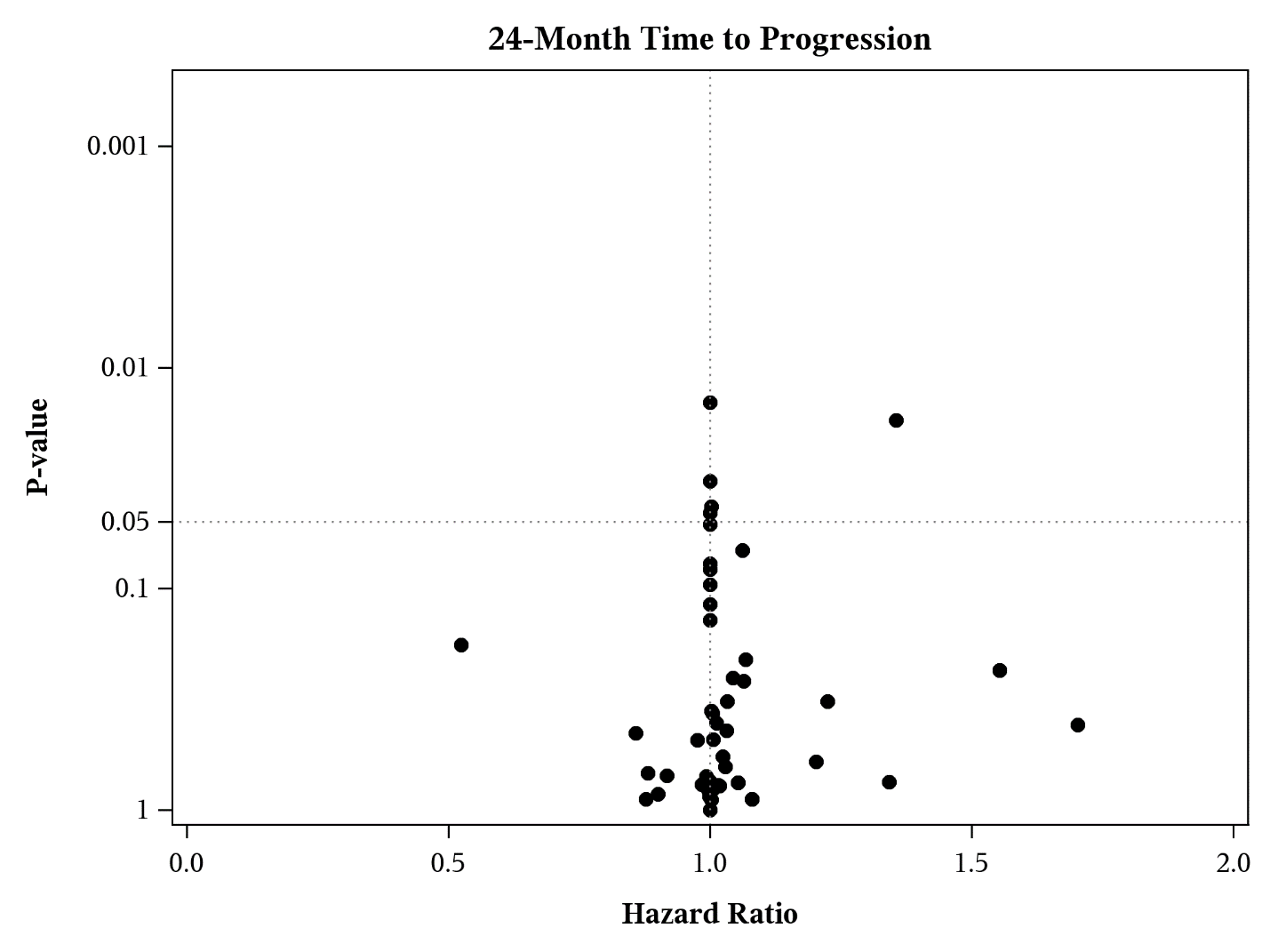


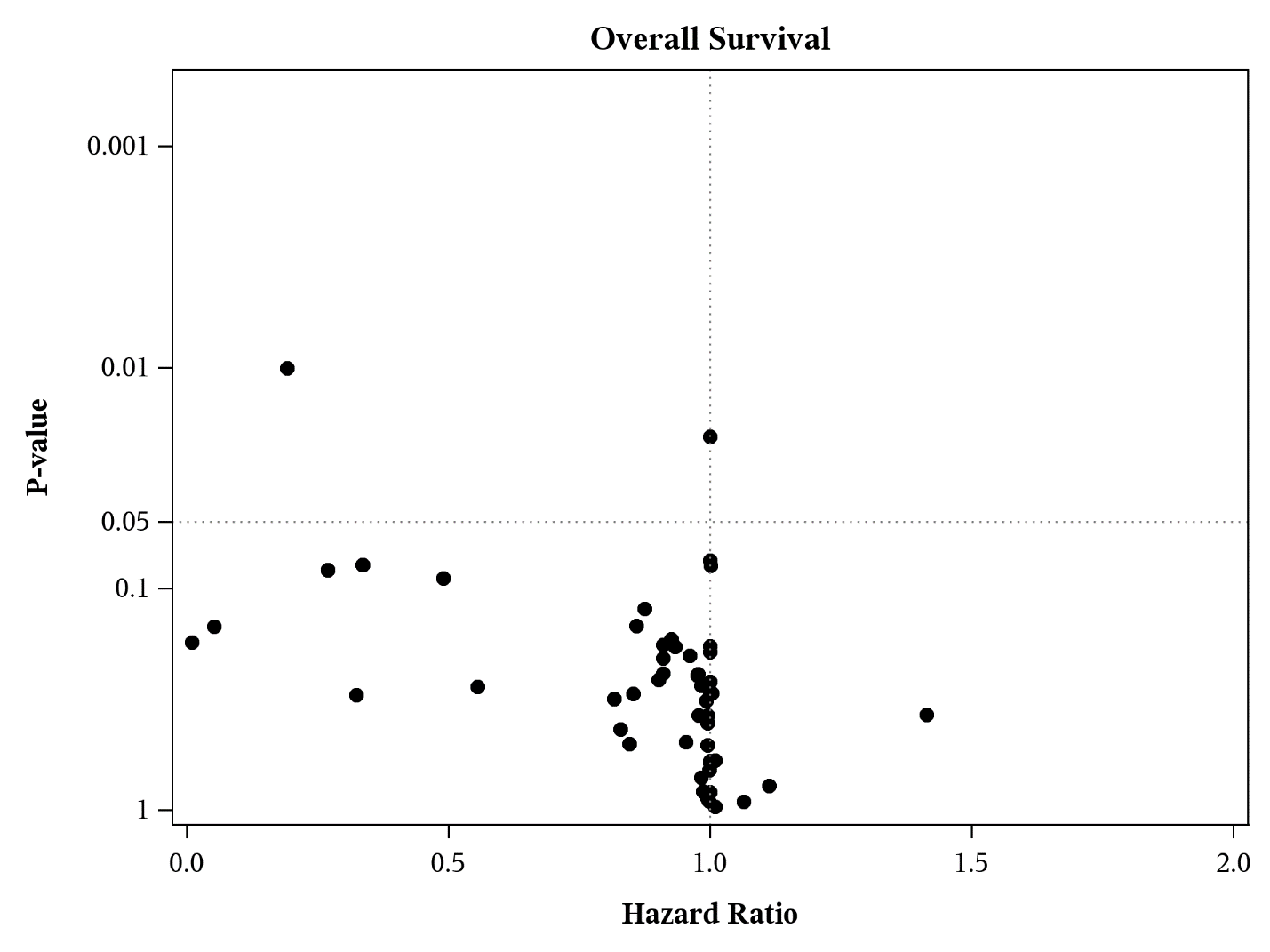


**Supplementary Figure-1.** Volcano plots of P-values and hazard ratios for 24-Month time to progression (TTP) and overall survival (OS). P-values are plotted on a logarithmic scale against the hazard ratio for each imaging feature. Significant imaging features are represented by dots above the dashed line at 0.05.

**Supplementary File-1.** PET/CT and acquisition and radiotracer injection parameters for all patients.
